# An Insight into Patients’ Perspectives of Ulcerative Colitis Flares via Analysis of Online Public Forum Posts

**DOI:** 10.1093/ibd/izad247

**Published:** 2023-11-02

**Authors:** David T Rubin, Joana Torres, Iris Dotan, Lan Terry Xu, Irene Modesto, John C Woolcott, Sean Gardiner, Bruce E Sands

**Affiliations:** Inflammatory Bowel Disease Center, University of Chicago Medicine, Chicago, IL, USA; Gastroenterology Division, Hospital Beatriz Ângelo, Loures, Portugal; Division of Gastroenterology, Hospital da Luz, Lisbon, Portugal; Faculty of Medicine, University of Lisbon, Portugal; Division of Gastroenterology, Rabin Medical Center, Petah Tikva, Israel; Sackler Faculty of Medicine, Tel Aviv University, Tel Aviv, Israel; Netbase Quid^™^, Santa Clara, CA, USA; Pfizer Inc, New York, NY, USA; Pfizer Inc, Collegeville, PA, USA; Pfizer Inc, New York, NY, USA; Dr. Henry D. Janowitz Division of Gastroenterology, Icahn School of Medicine at Mount Sinai, New York, NY, USA

**Keywords:** ulcerative colitis, flare, online forum, natural language processing

## Abstract

**Background:**

The knowledge of patients’ perceptions of factors contributing to ulcerative colitis (UC) flares is limited; however, online patient communications could offer insight. This analysis aimed to identify the most frequent patient-reported triggers and symptoms of UC flares, which could highlight potential interventions for outcome improvement.

**Methods:**

Online posts written pre- and postflare by patients with UC on 8 public forums in 6 countries between January 1, 2019, and February 14, 2021, were identified using flare-related keywords. Flare-related posts were captured and Netbase Quid^™^ artificial intelligence text analytics and natural language processing software were used to semantically map and identify commonly discussed themes and topics (subsets of themes).

**Results:**

Of >27 000 patient posts, 12 900 were identified as flare related. The most frequent themes were treatment experiences and side effects (28.5% of posts), followed by flare symptoms (22.9% of posts). The most frequent topic was emotional/peer support (9.4% of posts), followed by experiences with mesalamine (and other oral/rectal formulations; 8.0% of posts), and dietary recommendations (6.0% of posts). Stress and anxiety were the most frequently reported flare triggers (37.9% of posts), followed by diet (28.4% of posts). Stress and anxiety were frequently identified as both triggers for, and general symptoms of, flare. Blood in the stool was the most discussed flare indicator (57.8% of posts).

**Conclusions:**

Frequently discussed patient-perceived triggers of UC flares included diet, stress, and anxiety. These results suggest that physicians could incorporate a broader and more holistic approach to UC monitoring and management than is currently practiced.

**Key Messages**

- **What is already known?** Prediction of relapses and flares in ulcerative colitis (UC) has been extensively investigated using clinical parameters and biomarkers.
- **What is new here?** Analyses of flare-related language from UC online patient forum posts revealed that the most frequently discussed patient-perceived triggers of flare (diet, stress, and anxiety) are not routinely monitored during UC management.
- **How can this study help patient care?** These results highlight additional needs within UC management, research, and education and emphasize the need for physicians to incorporate a broader, more holistic approach to UC monitoring and management than currently practiced.

## Introduction

Ulcerative colitis (UC) is a chronic inflammatory condition characterized by periods of relapse and remission.^1^ Relapse of disease (objective recurrence or persistence of inflammation) or flare (patient-reported symptoms) can have a significantly negative impact on patients’ lives, both physically and psychologically, affecting work and life choices.^2^ In clinical practice, relapses and flares in UC are often assessed through a combination of patient-reported signs and symptoms, endoscopy, and measuring biomarkers of inflammation. Additionally, in clinical trials, flares are identified using scoring systems such as the Mayo score, the UC Endoscopic Index of Severity, and the Simple Clinical Colitis Activity Index.^1,3^ These scoring systems assess patient-reported clinical symptoms, such as stool frequency and rectal bleeding, and physician-reported endoscopic abnormalities, such as ulceration, erythema, and decreased vascular pattern.^1,3^

Few studies have investigated what patients perceive to be the triggers of UC flares. Identifying triggers of flare remains a difficult challenge due to lack of understanding of the mechanisms underlying a flare, the contributing factors, and discrepancies between relapses, as measured by objective inflammatory markers and flares as reported by patients.^4-6^ In addition, previous surveys of patients with UC and healthcare professionals (HCPs) have identified disparities in what each group perceived to be the most common flare trigger^2,7,8^ and the most important aspect of UC management.^2^ Clearer identification of predictors or triggers of flare in UC would allow clinical tailoring of patients’ education and care preflare or in the early stages of a relapse or flare, which could potentially improve treatment outcomes. While prediction of relapses and flares in UC has been extensively investigated using clinical parameters and biomarkers, such as C-reactive protein and fecal calprotectin,^9-12^ analyses of flare-related language from patients are rare.

Natural language processing (NLP) is a field of artificial intelligence (AI) that is often utilized to classify text data; it allows for the processing and analysis of large amounts of natural language data.^13^ NLP algorithms have previously been utilized to gain more insight into various aspects of inflammatory bowel disease (IBD), including patients’ understanding of the risks and benefits of biological therapies and injection-site reactions via social media posts,^14,15^ and potential associations between treatments and extraintestinal manifestations,^16^ the presence and status of extraintestinal manifestations,^17^ and the identification of surveillance colonoscopies through electronic medical records.^18^ This analysis utilized AI text analytics and NLP software to identify the most prevalent themes and topics (subsets of themes) among online public forum posts written by patients discussing UC flares, as well as self-reported triggers and symptoms of flares, which could highlight potential methods of intervention for flare mediation and outcome improvement.

## Methods

### Data Sources and Collection

Online posts written by patients with UC on 8 public UC forums in 6 countries between January 1, 2019, and February 14, 2021, were reviewed: Healing Well (United States), Crohn’s Forum (United States), Patient (United Kingdom), Educainflamatoria (Spain), Crohn Club Forum (Italy), Deutsche Morbus Crohn/Colitis ulcerosa Vereinigung e.V. (Germany), Afa Crohn RCH France (France), and Carenity (France, Germany, Italy, Spain, United Kingdom, and United States). First, using Netbase Quid^™^ (Santa Clara, CA, United States)AI text analytics and NLP software, online posts were identified as flare-related by the inclusion of certain keywords, which included explicit flare terminology (eg, flare and remission), potentially common UC symptoms (eg, cramps and diarrhea)^1^ and ontology building words that were not searched for individually but paired (eg, bloody poop, blood in stool). All words used to identify flare-related posts in this study can be found in Supplementary Table 1. Once identified, all flare-related posts were analyzed, categorizing each post into overarching themes, and mapped semantically. Posts written by the same author were also separated by date into 2 periods: preflare (ie, those posts written before the first mention of a flare) and postflare (ie, those written after the first mention of a flare).

### Outcomes

Details on the interpretation of network diagrams can be found in the Supplementary Methods. Data were mapped and ranked to identify the most prevalent themes and topics (subsets of themes) regarding flares discussed by patients with UC, overall and also stratified by region (posts by patients residing in the United States vs posts by patients residing in European countries [France, Germany, Italy, Spain, and the United Kingdom]). In addition, data were analyzed to examine patient-reported triggers and symptoms of flares and to evaluate treatment change behaviors.

Themes and flare triggers and symptoms were analyzed by total number of posts. Topics were either analyzed by total number of posts or ranked based on combined weighted scores from 5 quantitative metrics: the total number of posts within a topic (volume; 40%), the number of unique authors per topic (unique authors; 20%), the general attitude (positive or negative) of words within the posts of a topic (negative sentiment; 15%), the degree to which language within a topic was similar to other areas within the cluster network (betweenness centrality; 15%), and the average date of the posts (recency; 10%). Metrics are usually given the same weight. In situations where this is not the case, the volume of posts is generally given higher weight than the number of unique authors, as the volume of posts is the baseline metric that determines how often a topic/symptom/side effect is discussed. Negative sentiment was assessed using a “bag-of-words” model, which utilized a dictionary containing a set of words and a corresponding score. The model iterated through each word in a post and kept a running total of the sum of the scores. Treatment-change behaviors were evaluated based on a subset of flare-related posts that also discussed changing treatment (dose change and/or treatment switching).

## Results

### Patient Perceptions of UC Flares

#### Frequently discussed themes and topics

**Figure 1.**
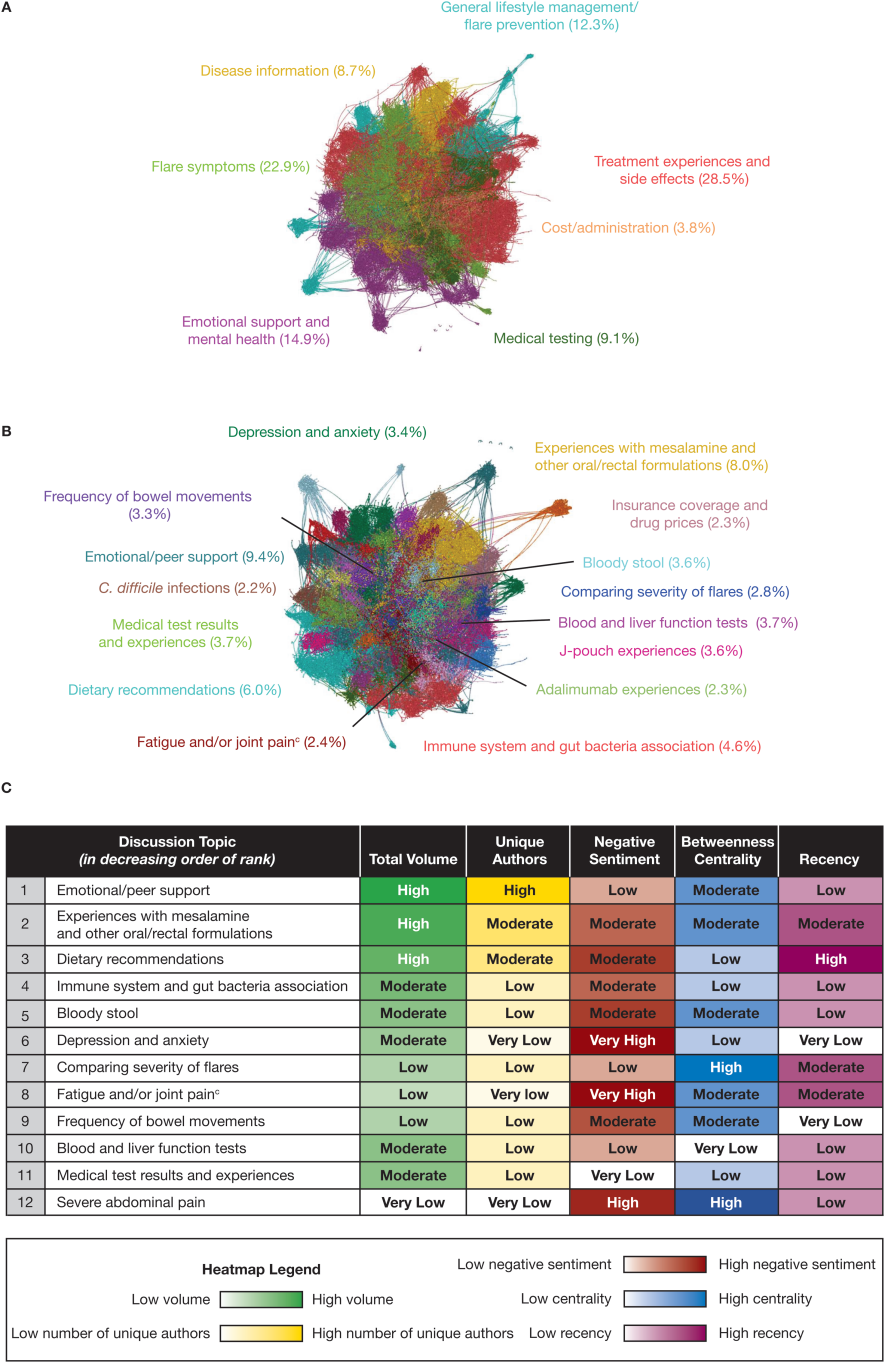
(A) The most frequently discussed themes in flare-related posts by patients with ulcerative colitis,^a^ (B) the most frequently discussed flare-related topics by patients with ulcerative colitis,^a^ and (C) the top 12 ranked flare-related topics discussed by patients with ulcerative colitis.^b^ Flare-related posts, including those from the same author pre- and postflare, were identified from posts written by patients with ulcerative colitis on 8 online public forums in 6 different countries due to the inclusion of keywords. Data were then mapped semantically. ^a^Flare-related post data were color-coded by themes and topics; each node represents a post, and connections represent similar language used across the posts. Centrally located nodes represent core concepts and peripheral nodes represent niche concepts. Percentages represent total volume of posts. ^b^Top topic rankings are a result of a combined weighted score of volume (40%), unique authors (20%), negative sentiment (15%), betweenness centrality (15%), and recency (10%). ^c^Joint pain and fatigue were analyzed together due to the proportion of posts that mentioned both symptoms (approximately 20% of the 2.4% of posts). *C. difficile*, *Clostridioides difficile*.

Of >27 000 patient posts from 1876 unique authors in the selected online patient forums on UC, 12 900 were identified as flare-related. The most frequent themes identified within the patient flare-related posts were treatment experiences and side effects (28.5% of posts) and flare symptoms (22.9% of posts) (Figure 1A), which together accounted for half of all flare-related posts. Other flare-related themes included emotional support and mental health (14.9% of posts), general lifestyle management/flare prevention (12.3% of posts), medical testing (9.1% of posts), disease information (8.7% of posts), and cost/administration (3.8% of posts) (Figure 1A).

The most frequently discussed topics were emotional/peer support (9.4% of posts), followed by posts on experiences with mesalamine (and other oral/rectal formulations; 8.0% of posts), which was the most frequently discussed treatment option, and dietary recommendations (6.0% of posts) (Figure 1B). Of the top 12 ranked topics (as determined by the 5 quantitative metrics) identified within the flare-related posts, 8 had moderate to very high negative sentiment scores (Figure 1C); most posts were from patients whose disease was not adequately controlled and who wanted to discuss this with other patients with UC. Topics with high/very high negative sentiment scores were severe abdominal pain (high), depression and anxiety (very high), and fatigue and/or joint pain (very high) (Figure 1C). Due to these high negative sentiment scores, fatigue and/or joint pain and severe abdominal pain were ranked in the top 12 topics, despite having low and very low total post volume, respectively (Figure 1B and 1C).

#### Patient-perceived triggers of UC flares

Among posts related to frequently self-reported flare triggers (N = 1161), a period of stress and anxiety was the most frequently perceived trigger of UC flares (37.9% of posts) (Table 1). The external stressors attributed to triggering a flare were generally situational, such as a divorce, death, or a work/school issue. Some patients described a belief that their flares would cause anxiety and depression due to the perceived impact of UC and the biological impact of gut bacteria on mental health. Patients discussed how these feelings of anxiety and depression would feed back into feelings of stress, resulting in a negative cycle that would exacerbate their symptoms, thus proving difficult to manage. During these times, patients often struggled to distinguish whether anxiety and/or stress were the cause or an effect of their perceived flare.

**Table 1.** Most frequently discussed flare triggers and initial flare symptoms in patients with UC (total volume of posts).

Order	Flare triggers (% of posts) (N = 1161)	Initial flare symptoms (% of posts) (N = 645)
1	Stress and anxiety (37.9%)	Blood in stool/passing blood (57.8%)
2	Diet (28.4%)^a^	Diarrhea and loose stool (19.1%)
3	Smoking cessation (9.0%)	Stool frequency (18.6%)
4	Antibiotics (8.9%)	Mucus in stool/passing mucus (17.1%)
5	Bacterial/viral infection (7.1%)^b^	Pain and cramping (13.9%)
6	NSAID usage (5.7%)	Fatigue (7.0%)
7	Medical procedures (<5.0%)	Bloating/feeling gassy (5.6%)
8	Hormonal changes (<5.0%)	Rash (<5.0%)
9	Lack of sleep (<5.0%)	Migraine/headache (<5.0%)
10	Running/intense exercise (<5.0%)	Fever/high temperature (<5.0%)

Flare-related posts, including those from the same author pre- and postflare, were identified from posts written by patients with UC on 8 online public forums in 6 different countries due to the inclusion of keywords. Flare triggers and initial symptoms (experienced at the start of a flare) were identified and ordered by the total volume of posts.

Abbreviations: N, total number of posts; NSAID, nonsteroidal anti-inflammatory drug; UC, ulcerative colitis.

^a^Common foods that were perceived to trigger flares included dairy, alcohol, gluten, coffee, meat, spicy food, artificial sugar, and high-carbohydrate and high-sugar foods.

^b^Patients who identified bacterial/viral infections as a flare trigger had uniquely low confidence and were unsure about infections being the cause of their triggers.

Diet was the second most frequently perceived trigger of flares (28.4% of posts) (Table 1). In 68.0% of flare-related posts discussing diet, patients described their diet as playing a key part in maintaining remission but believed that flares were caused by a variety of factors. In contrast, in 32.0% of flare-related posts discussing diet, patients believed that their flares had been caused by a change in their diet.

#### Patient-perceived initial symptoms of UC flares

In most of the posts related to flare management (60.2% [N = 4042]), patients monitored their symptoms (absence, presence, and severity) in an attempt to understand and manage their flares, while in the remaining posts (39.8%), patients placed additional importance on clinical tests. However, among posts related to identifying flares (N = 645), patients did not commonly make a distinction between initial (experienced at the start of a flare) or general (experienced throughout a flare) symptoms when discussing their perceived flare symptoms. Blood in the stool was the most frequently discussed flare indicator (experienced at the start of a flare; 57.8% of posts [N = 645]) and was often co-mentioned with mucus in the stool, followed by diarrhea and loose stools (19.1% of posts), and increased frequency of bowel movements (stool frequency; 18.6% of posts) (Table 1). Patients reported that they often confused blood in the stool with hemorrhoidal bleeding, and also reported diarrhea as a common symptom for gastrointestinal infections and dietary intolerances, which resulted in a delay in seeking medical advice for some patients (28.2% of posts from patients with blood in their stool and 83.4% of posts from patients with diarrhea).

### Treatment-Change Behaviors

**Figure 2.**
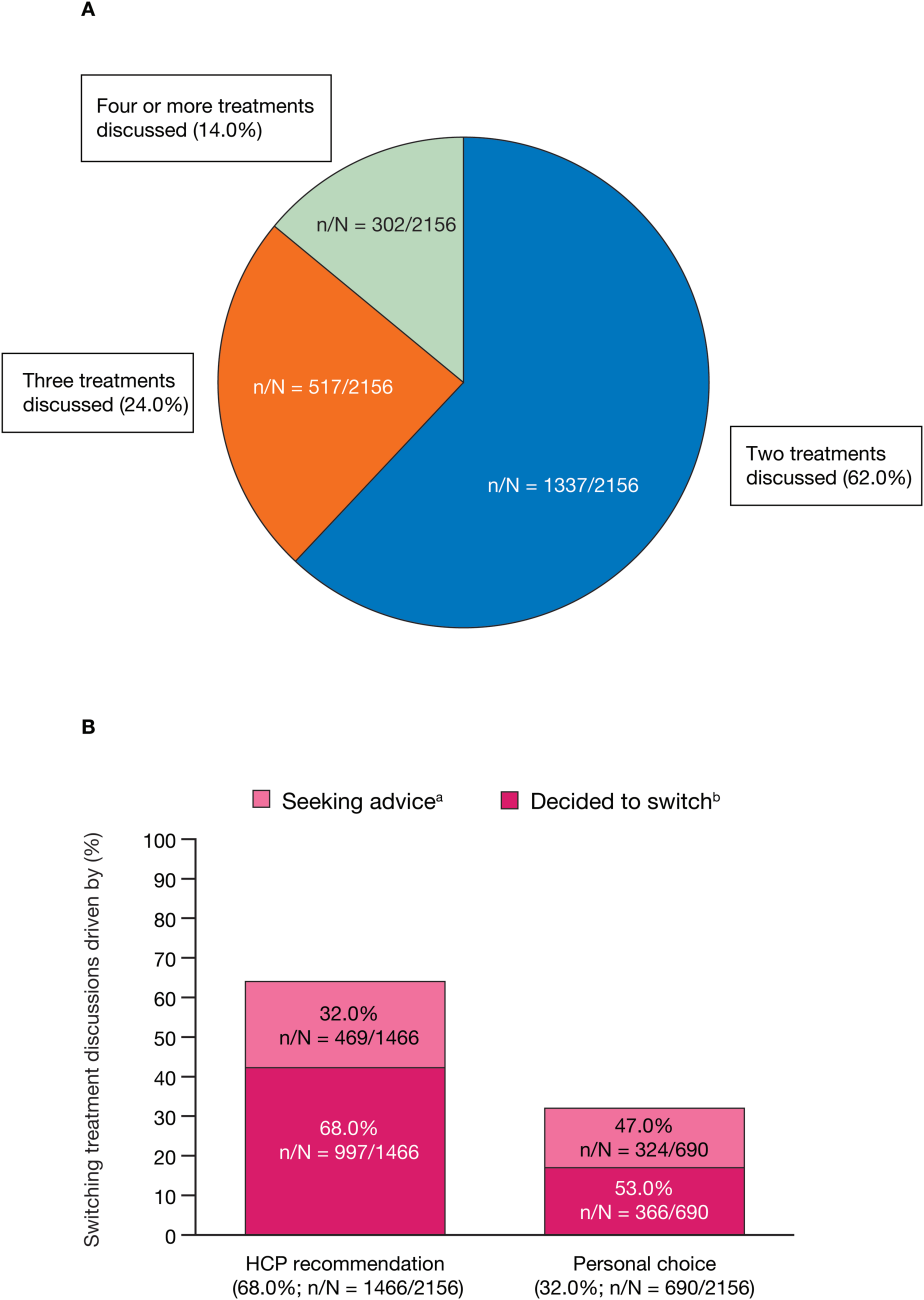
(A) Proportions of flare-related posts discussing treatment efficacy by the number of treatments discussed and (B) the drivers of treatment-switching decisions. Posts (N = 2156) are from patients who discussed flares and treatment switching on 8 online public forums in 6 different countries. ^a^Posts were from patients who were seeking advice and asking for more information about whether they were making the right choice. ^b^Posts were from patients who had decided to switch and were discussing pre- and postswitch experiences. HCP, healthcare professional; n, number of posts in each category; N, total number of posts.

Patients discussing flares also voiced their frustrations about trying to find the right treatment for UC. Of those posts discussing flares that also discussed switching treatment (N = 2156), 38.0% mentioned that patients had switched on 3 or more occasions (Figure 2A). The most frequently discussed reasons for switching treatments were side effects or intolerance (50.0% of posts), inefficiency/disease progression (34.9% of posts), cessation of prior efficacy (19.9% of posts), and the worry about long-term effects of treatment (7.5% of posts) (Table 2). When discussing these reasons for switching treatment within flare-related posts, few patients discussed changes in dosage, and none of the patients switching treatment due to a cessation of efficacy discussed dosage change. Of those flare-related posts that did discuss dosage change (N = 859), the majority of discussions were about decreasing vs increasing dosage (post volume of 94.9% vs 5.1%, respectively). The most frequently discussed reasons for a decrease in dose were clinical guidance/tapering (89.3% of posts discussing a decrease in dose, with prednisone being the most discussed treatment that was decreased in dose) and side effects (3.5% of posts discussing a decrease in dose); the most frequently discussed reasons for an increase in dose were limited efficacy (43.2% of posts discussing an increase in dose) and a switch in long-term maintenance treatment due to an active flare (29.5% of posts discussing an increase in dose) (Table 2). Of those posts discussing flares and switching treatment decisions after remission (N = 385), 94.2% discussed continuing maintenance therapy (often with a lower dose of the current treatment), which was due to HCP recommendations and/or the fear of recurrence. The remaining 5.8% of posts discussed stopping treatment, which was due to fear about the long-term effects of treatment, or because they had previously been in remission for an extended time, or because they hoped to manage remission through lifestyle changes.

**Table 2.** Most frequently discussed reasons for treatment switching or change of dose (total volume of posts).

Order	Switching treatment (N = 2156)	Increase in dose (relative volume of posts) (N = 44)	Decrease in dose (relative volume of posts) (N = 815)
1	Side effects or intolerance (50.0%)	Limited efficacy (43.2%)	Clinical guidance/tapering (89.3%)
2	Inefficacy/disease progression (34.9%)	Switch from long-term maintenance treatment due to active flares (29.5%)	Side effects (3.5%)
3	Cessation of prior efficacy (19.9%)	NR	NR
4	Worry about long-term effects (7.5%)	NR	NR

Flare-related posts, including those from the same author pre- and postflare, were identified from posts written by patients with UC on 8 online public forums in 6 different countries due to the inclusion of keywords. Reasons for treatment switching or change of dose were identified and ordered by the total volume of posts.

Abbreviations: N, total number of posts; NR, not reported; UC, ulcerative colitis.

Of the flare-related posts discussing switching treatments (N = 2156), 68.0% described the decision to switch as being driven by HCP recommendations, while 32.0% discussed switching due to personal choices, such as experiences of drug intolerances, fear of long-term effects of certain treatments, and personal finances (Figure 2B). The proportion of posts suggesting that the decision to switch had already been made was higher for decisions driven by HCPs vs those driven by a personal choice (68.0% vs 53.0% of posts, respectively) (Figure 2B).

### Regional Differences in Flare-Related Discussion Themes and Topics

**Figure 3.**
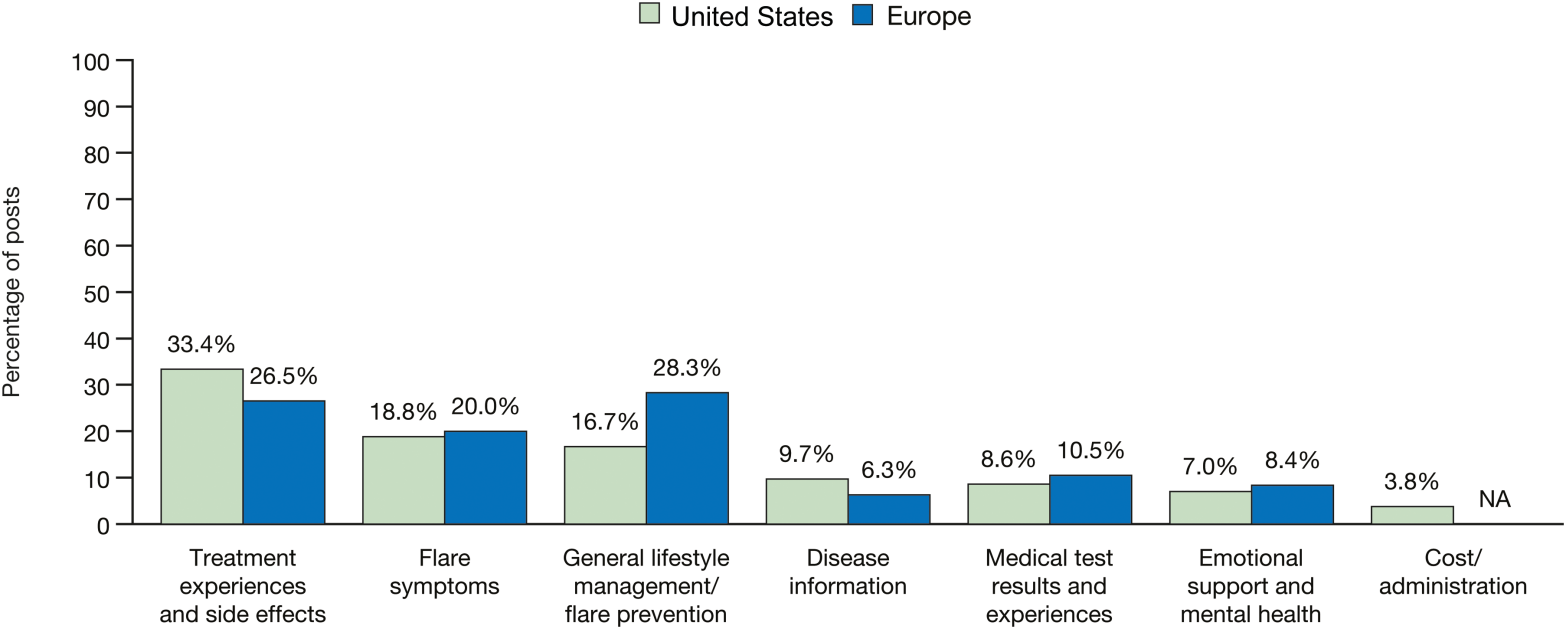
The most frequently discussed themes in flare-related posts by patients with ulcerative colitis in the United States and Europe. Flare-related posts, including those from the same author pre- and postflare, were identified from posts written by patients with ulcerative colitis on 8 online public forums in 6 different countries due to the inclusion of keywords. Posts were stratified by region (United States and European countries [France, Germany, Italy, Spain, and the United Kingdom]) and discussion themes were identified and ordered by total volume of posts. NA, not applicable.

The overarching UC flare-related discussion themes were similar in content between posts from the United States (N = 10 284) and Europe (N = 2567), with the exception of cost/administration, which was unique to the United States (Figure 3). Treatment experiences and side effects was the most frequently discussed theme in flare-related posts written by patients in the United States (33.4% of posts), and general lifestyle management/flare prevention was the most frequently discussed theme for patients in Europe (28.3% of posts) (Figure 3).

**Figure 4.**
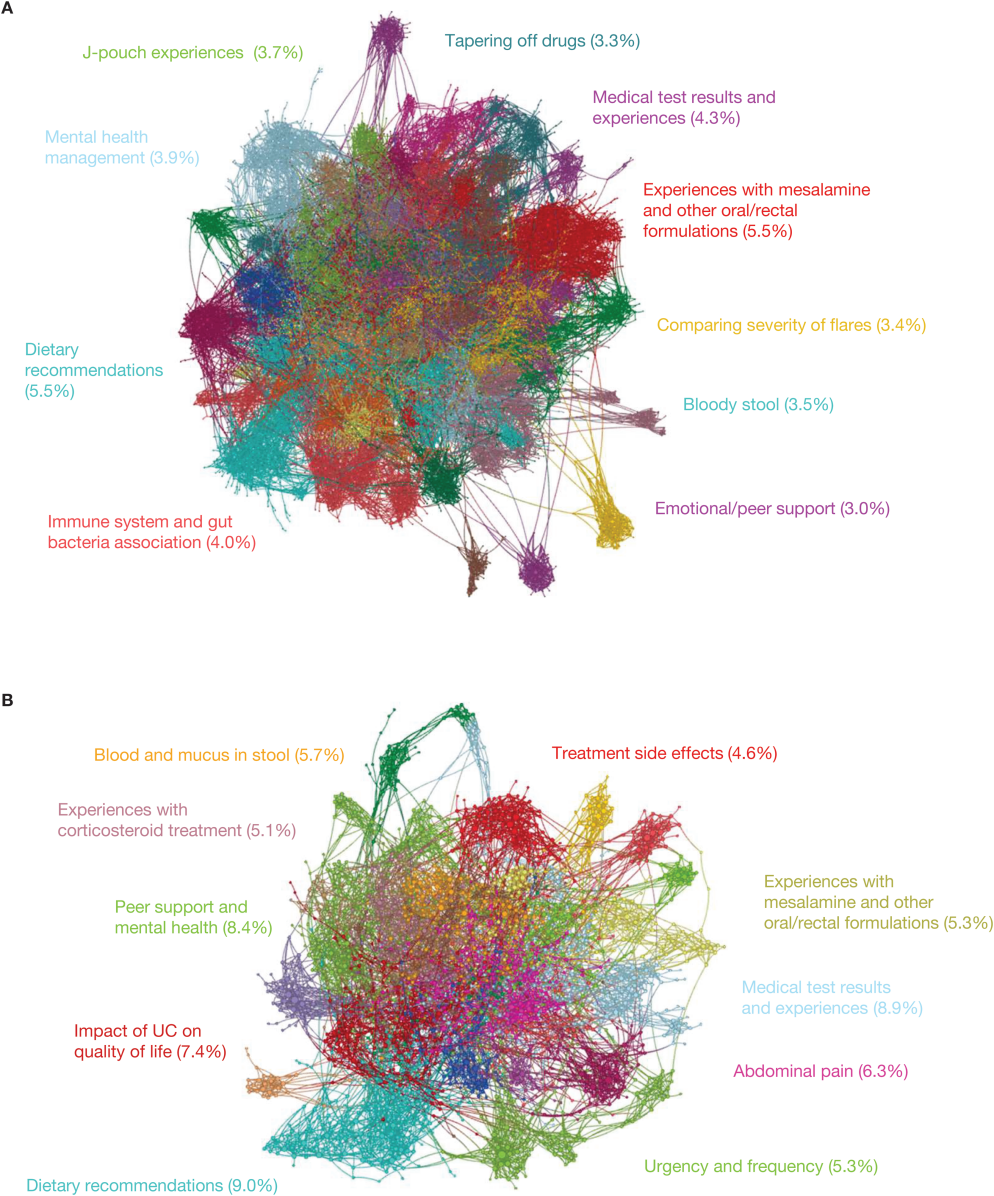
The most frequently discussed flare-related topics by patients with ulcerative colitis (A) in the United States and (B) in European countries. Flare-related posts, including those from the same author pre- and postflare, were identified from posts written by patients with ulcerative colitis on 8 online public forums in 6 different countries due to the inclusion of keywords. Posts were stratified by region (United States and European countries [France, Germany, Italy, Spain, and the United Kingdom]) and color-coded by topics; each node represents a post, and connections represent similar language used across the posts. Centrally located nodes represent core concepts and peripheral nodes represent niche concepts. Percentages represent total volume of posts.

Experiences with mesalamine and other oral/rectal formulations (5.5% of posts), J-pouch experiences (experiences with J-pouch surgery, postoperative care, and pouchitis; 3.7% of posts), and tapering off drugs (3.3% of posts) were the most frequent treatment-related topics discussed among patients in the United States (Figure 4A). In comparison, lifestyle and support were the most frequent topics of flare-related discussions had by patients from Europe, including dietary recommendations (9.0% of posts), peer support and mental health (8.4% of posts), and impact of UC on quality of life (7.4% of posts) (Figure 4B).

The flare-related topics that were ranked within the top 10 (as determined by the 5 quantitative metrics) for both the United States and European countries included: (1) dietary recommendations, (2) mental health (mental health management [United States]; peer support and mental health [Europe]), (3) medical test results and experiences, and (4) flare symptoms (bloody stool [United States], blood and mucus in stool [Europe], and urgency and frequency [both United States and Europe]) (Table 3 and Supplementary Figure 1). Of those flare-related topics that were uniquely discussed by patients from the United States, the top 3 were tapering off drugs, comparing the severity of flares and the different treatment options for each stage, and experiences with budesonide. For patients in Europe, the top 3 unique flare-related discussion topics were the overarching impact of UC on quality of life, experiences with corticosteroid treatment and corticosteroid treatment failure, and proctitis diagnosis and treatment, particularly mesalamine rectal formulations.

**Table 3.** The top 10 ranked flare-related topics discussed by patients with UC, by geographic region.

Ranking	U.S. topics	European topics
1	Dietary recommendations^a^	Dietary recommendations^a^
2	Experiences with mesalamine and other oral/rectal formulations	Peer support and mental health^a^
3	Medical test results and experiences^a^	Impact of UC on quality of life^b^
4	Immune system and gut bacteria association	Medical test results and experiences^a^
5	Mental health management^a^	Abdominal pain
6	Bloody stool^a^	Blood and mucus in stool^a^
7	Tapering off drugs^c^	Experiences with corticosteroid treatment
8	Comparing severity of flares	Urgency and frequency^a^
9	Urgency and frequency^a^	Treatment side effects
10	Experiences with oral and rectal budesonide formulations^d^	Impact of antibiotics on UC flares

Flare-related posts, including those from the same author pre- and postflare, were identified from posts written by patients with UC on 8 online public forums in 6 different countries due to the inclusion of keywords. Posts were stratified by region (United States and European countries [France, Germany, Italy, Spain, and the United Kingdom]) and topics were identified and ranked by a combined weighted score of volume (40%), unique authors (20%), negative sentiment (15%), betweenness centrality (15%), and recency (10%).

Abbreviation: UC, ulcerative colitis.

^a^Common topics in U.S. and European discussions.

^b^Particularly in the workplace.

^c^Patients shared tapering schedules and expressed concerns over flare recurrence.

^d^Foam enemas had a more positive sentiment than extended-release oral formations.

## Discussion

In this analysis of patients with UC posting on online public disease forums, advanced AI text analytics and NLP were leveraged to identify the most prevalent themes and topics discussed in relation to disease flares. Of note, half of the flare-related discussions were related to treatment experiences and side effects and flare symptoms, and a quarter were related to emotional support and lifestyle management.

Given that UC is a complex intestinal disease with interplaying genetic, environmental, and biological (microbial and immunological) factors contributing to its pathogenesis,^4,19,20^ it was not surprising that dietary recommendations were commonly mentioned by patients with this condition (6.0% of flare-related posts). The results of this analysis suggest that patients perceive diet to be a major environmental factor, with a controlled diet being associated with the maintenance of remission, and changes in diet with triggering flares. Consistent with these results, in recent analyses of social media posts by patients with IBD, diet and nutrition were among the top topics of interest.^21,22^ The impact of diet and nutrition on UC flares remains an area that requires further research and confirmation to inform dietary recommendations in clinical guidelines and practice. The PREdiCCt (PRognostic effect of Environmental factors in Crohn’s and Colitis) study (NCT03282903) of patients with IBD in clinical remission will collect information on diet, lifestyle, and gut microbiome over 2 years to investigate any potential associations with flare symptoms. At the baseline of the PREdiCCt study, dietary change was one of the most common patient-reported causes of flare.^23^

Patients’ mental well-being is another environmental factor proposed to contribute to UC pathogenesis and was highly discussed in flare-related posts in this analysis. Patients clearly perceived stress, anxiety, and depression as major contributors to their self-reported flares. Patients identified these factors as both triggers and symptoms of flares and described themselves as becoming stuck in a negative cycle of stress, anxiety, and disease activity. This perception by patients is consistent with previous research showing anxiety and depression as common comorbidities of IBD that are experienced at higher rates during active disease.^24,25^ Furthermore, there is evidence to support that stress not only is a psychological response, but also is a biological response.^26^ The results of this analysis reinforce the need for physicians to incorporate a broad approach when monitoring UC, as well as a need for further tools to monitor and manage stress, anxiety, and depression. Furthermore, these findings emphasize that more weight should be given to the patient’s perception of their mental health status, regardless of clinical assessment. Previous studies have reported associations between perceived high levels of stress and increased risk of flares (based on patient-reported symptoms) in patients with IBD.^27,28^ The evidence presented here suggests that patients often attribute their self-reported flares to periods of stress in their lives, for example due to work and school. Taking an alternative prophylactic approach in treatment could potentially benefit those patients who are expecting stressful periods in their life and could give them the perception of control over their disease. However, there remains an important gap in the evidence; it is not known whether avoiding stress or treating anxiety or depression prevents relapses and/or flares, and similarly whether treatment of stress and mental health disorders would restore control of relapses and/or flares.

Many patients with UC may develop a complicated disease course in terms of lack or loss of response to treatments.^29^ Discussions on treatment experiences and side effects accounted for 28.5% of the flare-related posts analyzed in this analysis. Similarly, in a recent analysis of online social media posts discussing IBD and distress, “medication” was the second most mentioned topic.^21^ In this analysis, patients’ posts expressed frustrations when discussing attempts to find the right treatment for them, and 38% of posts discussing flares and switching treatments mentioned that patients had tried 3 or more different treatments. The bias in reporting data from patients who are not responding or who are not tolerating their current therapies is acknowledged; patients who have controlled disease may be less likely to be motivated to post on social media about their disease. However, there is still much to learn from these posts.

When describing treatment-switching decisions, the changes were mainly driven by HCP recommendations (68.0% of posts). Of those discussions on dosage changes, the vast majority of flare-related posts were in relation to decreasing doses (94.9%). In addition, 94.2% of posts from patients who achieved remission discussed continuation of maintenance therapy, which often consisted of lowering dosages. Interestingly, a large proportion of patient discussions in the United States were treatment focused (33.4% of posts), unlike discussions in European countries, which were almost equally focused on treatment experiences and side effects (26.5%) and general lifestyle management/flare prevention (28.3%). Furthermore, tapering off drugs and insurance coverage and drug prices were uniquely discussed by patients in the United States, raising the question as to whether treatment tapering was cost-related as opposed to being based on patient preferences.

In the posts evaluated in this analysis, patients were discussing their symptoms to try to understand their disease, were often seeking advice, and had less confidence when making their own decisions compared with their HCP’s judgement. The “crowdsourcing” use of social media and electronic health tools may increase patient and HCP interactions; studies have suggested that patients with IBD are receptive to their use in monitoring disease and interacting with HCPs.^30,31^ The use of electronic health tools, such as wearables, IBD-specific social media platforms, and mobile phone applications, may also help to improve monitoring of UC and potential flares.

The distinction between patient-reported flares and disease relapses should be further elucidated. Symptoms are directly related to quality of life and remain very important, but distinguishing symptoms from objective measures of disease control could be useful in future approaches to disease education and patient empowerment. If a patient experiences a diet-triggered symptom exacerbation but subsequently learns that there has been no worsening of inflammation, they may be able to manage their symptoms with dietary adjustments. However, a patient who has symptoms with subsequent identification of objective disease activity may require a different management strategy. This study identifies the important area of patient perceptions of their flares, and that perception can become a reality in the minds of individual patients. Further work to provide patients with additional insight into the disease process is expected to help address this important issue.

Strengths of this analysis include the fact that posts were made in patients’ native languages, which would have prevented miscommunication through the potential incorrect use of a second language and may have allowed a greater selection of vocabulary. A further strength was that this analysis was performed on patient posts in open-access online forums, which would have gathered more free and organic conversation, as opposed to potentially restricted conversation via directed questions conducted in patient surveys in research and clinical settings. In addition, posts from the same patient pre- and postflare were included in this analysis, which allowed a degree of temporal differentiation between triggers and symptoms.

Limitations of this analysis include its subjective nature—data were taken from the patients’ perspectives only, with no objective measure to confirm whether patients were experiencing flares according to standard clinical assessments. Therefore, it is possible that patient-reported flares were functional in nature. In addition, confirmation that posts were made by patients with UC was not obtained. A further limitation is the lack of longitudinal data on the length of time between posts and a patient-reported flare, which could be useful in elucidating whether there were any patterns in the order, length, and/or severity of symptoms. In addition, these discussions were biased towards patients who were comfortable sharing their experiences in online public forums and, as discussed previously, may not have included patients whose disease was mild or controlled; therefore, these data may not be representative of all patients with UC.

## Conclusions

Diet, stress, and anxiety, the most frequently discussed patient-perceived triggers of flares identified in this analysis, are not routinely monitored during UC management or built into standard goals of UC treatment.^1,32^ These results highlight additional needs within UC management, research, and education, in terms of a lack of understanding the difference between symptoms and disease activity, the role of diet in UC management, and additional guidance on continuous monitoring of a patient’s mental health. Further, the findings of this analysis emphasize the need for physicians, IBD nurses, and other members of IBD healthcare teams to incorporate a broader and more holistic approach to UC monitoring and management than currently practiced.

## Supplementary data

Supplementary data is available at *Inflammatory Bowel Diseases* online.

izad247_suppl_Supplementary_Material

## Data Availability

Upon request, and subject to review, Pfizer will provide the data that support the findings of this study. Subject to certain criteria, conditions, and exceptions, Pfizer may also provide access to the related individual de-identified participant data. See https://www.pfizer.com/science/clinical-trials/trial-data-and-results for more information.
