# Supplementary material for: An Insight into Patients’ Perspectives of Ulcerative Colitis Flares via Analysis of Online Public Forum Posts
Source: Inflamm Bowel Dis. 2023 Nov 2;30(10):1748–58. doi: 10.1093/ibd/izad247 (PMC11447010; doi:10.1093/ibd/izad247)
Supplement: izad247_suppl_Supplementary_Material [file izad247_suppl_supplementary_material.pdf]

Supplementary Materials

Methods

Interpretation of Network Diagrams

Netbase Quid™ artificial intelligence text analytics and natural language processing (NLP) software algorithms have previously been described and utilized to analyze patient perspectives and experiences of breast cancer, atopic dermatitis, and tardive dyskinesia.[1-3] In brief, within a network diagram, each patient post was represented by a node, and posts with similar language were clustered together and connected. Color-coded clusters of nodes represented the different topics discussed, and the density of the clusters indicated how similar or diverse the posts were within the cluster. The nodes/posts that were centrally located contained language that was shared across multiple clusters, and thus represented core concepts. The clusters on the periphery represented niche topics that were not representative of the larger landscape. Greater distances between the clusters indicated a low number of interrelated posts, and a bridging node between 2 clusters indicated a post at an intersection between 2 concepts. The NLP algorithm used the Louvain method[4] to group posts by common topics based on shared similar language. Themes were mapped using a combination of keyword identification and grouping with input from the analysis team. All proprietary treatment names were replaced with generic names.

Supplementary Table 1. Flare-related keywords.

Explicit Flare Terminology				
**English**	**Spanish**	**German**	**Italian**	**French**
Flare	Brote, recaída	Schub	Infiammazione, riacutizzazione, fase acuta	Poussée
Flared			Infiammata, infiammato, infammate, infiammati	
Flaring				
Flares			Infiammazioni, riacutizzazioni, fasi acute	
Flair				
Flaired				
Flairing				Poussée
Flairs				Flambée
Attack	Ataque	Attacke, fulminanter schub, heftigen schub	Attacco	Crise
Remission	Remisión	Remission, rückgang, vorübergehendes nachlassen	Remissione	Rémission
Relapse	Recaída	Rückfall, nachlass, reduzierung, minderung	Ricaduta, ripresa di malattia	Rechute

Inflammation	Inflamación	Entzündung	Infiammazione, flogosi	Inflammation
Active inflammation	Inflamación activa	Aktiv inflammation	Infiammazione attiva, flogosi attiva, flogosi presente	Inflammation active
Active disease	Enfermedad activa	Aktive erkrankung	Malattia attiva, patologia attiva	Une maladie active
Symptom	Síntoma	Symptom	Sintomo, sintomi, disturbo, disturbi	Symptôme, symptômes
Acute phase		Akutphase		

Potential, Common Ulcerative Colitis Symptoms[a]

English	Spanish	German	Italian	French
Bloating, bloated, bloat	Distensión abdominal, hinchado, hinchazón, distendido	Blähungen, aufgebläht, aufblasen	Gonfiore, gonfio, meteorismo	Ballonnements, ballonné
Swollen stomach	Estómago hinchado, estómago inflamado, barriga hinchada, tripa hinchada	Geschwollener bauch	Gonfia lo stomaco, gonfiore di stomaco, stomaco gonfio	Estomac gonflé
Cramp, cramps, cramping, cramped	Calambres, cólicos	Krampf, krämpfe, krämpfe, beengt	Crampo, crampi, crampiforme	Crampes, des crampes, des crampes
Spasm, spasms	Espasmo, espasmos	Krampf, krämpfe	Spasmo, spasmi, spastico	Spasmes, spasmes
Pain, painful	Dolor, doloroso	Schmerzen, schmerzen	Dolore, dolori, doloroso	Douleur, douloureux
Hurt, hurts	Dolor, duele	Schmerz, verletzt	Male, fa male, avere male, avere dolore	Mal, ça me fait mal

Stomachache	Dolor de estómago, dolor de tripa, dolor de barriga	Magenschmerzen	Mal di stomaco, dolore allo stomaco, crampi allo stomaco, fitte allo stomaco	Maux d'estomac, avoir mal au bide, douleurs abdominales
Uncomfortable, discomfort	Incómodo, molestias, incomodidad, disconfort	Unbequem, beschwerden	Disagio, a disagio	Mal à l'aise, l'inconfort, malaise
Tender, tenderness	Sensible, sensibilidad	Zärtlich, zärtlichkeit	Dolorabile alla palpazione, dolorabilità al tatto	Sensibles, très sensibles, sensibilité
Diarrhea	Diarrea	Durchfall	Diarrea, dissenteria	La diarrhée, diarrhées
Watery stool	Heces líquidas, deposiciones líquidas, evacuaciones líquidas	Wässriger stuhl	Diarrea acquosa, feci liquide	Selles liquides
Loose stool	Heces sueltas, heces blandas	Weicher stuhl	Feci morbide, feci molli, feci mollicce	Selles molles
Stool with mucus	Heces con moco, deposiciones con moco, evacuaciones con moco	Hocker mit schleim	Feci con muco, diarrea con muco, mucorrea	Selles avec du mucus
Stool with pus	Heces con pus, deposiciones con pus, evacuaciones con pus	Hocker mit eiter	Feci con pus, feci purulente, feci mucopurulente, diarrea con muco e pus	Selles purulentes
Bloody stool	Heces con sangre, deposiciones con sangre, evacuaciones con sangre, deposiciones o evacuaciones sanguinolentas	Blutiger stuhl	Feci con sangue, diarrea con sangue, ematochezia	Selles sanglantes
Blood	Sangre	Blut	Sangue	Du sang

Rectal bleeding	Sangrado rectal	Rektale blutung	Sanguinamento rettale, proctorragia, rettoragia, evacuazioni mucoematiche	Saignement rectal
Frequency, frequent	Frecuencia, frecuente	Frequenz, häufiger	Frequenza, frequente	Fréquence, fréquente
Urgency, urge	Urgencia, urgente	Dringlichkeit, drang	Urgenza, avere urgenza, scappare in bagno	Urgence, envie
Incontinence	Incontinencia	Inkontinenz	Incontinenza, non trattenere, farsela addosso	Incontinence
Constipation, constipated	Estreñimiento, extreñido, constipación, constipado	Verstopfung, verstopfung	Costipazione, costipato, stitico, stitichezza	Constipation, constipé
Gas	Gas, gases	Gas	Gas, aria	Gaz
Flatulence	Flatulencia, gases	Blähung	Flatulenza	Flatulence
Fart	Pedo, se tiró un pedo, flato	Furz, gefurzt	Scoreggia, puzzetta, peto, gas intestinali, gas intestinale	Péter, pété
Borborygmi	Borborigmos, ruidos intestinales, ruidos en la barriga o tripa	Borborygmi	Borborigmi, movimento di aria nella pancia, rumori della pancia	Borborygmes
Body ache	Dolor corporal, dolor en el cuerpo	Körperschmerzen	Dolore diffuso, dolenzia del corpo, dolore ovunque, male ovunque, dolori in tutto il corpo, malessere generale	Courbatures
Tender joints	Articulaciones sensibles, dolor articular, articulaciones dolorosas	Ausschreibung gelenke	Articolazioni dolorose al tatto, articolazioni dolenti al tatto, articolazioni dolorabili al tatto	Articulations sensibles, douleuresues

Painful joints	Articulaciones dolorosas, dolor en las articulaciones	Schmerzende gelenke	Articolazioni dolorose, articolazioni dolenti	Articulations douloureuses
Swollen joints	Articulaciones inflamadas	Geschwollene gelenke	Gonfiore delle articolazioni, articolazioni gonfie	Gonflement des articulations
Back pain	Dolor de espalda	Rückenschmerzen	Mal di schiena, male alla schiena, dolore alla schiena	Mal au dos, mal de dos
Backache	Dolor de espalda	Kreuzschmerzen	Lombalgia	Mal au dos
Red eyes	Ojos rojos	Rote augen, augenentzündung	Occhi rossi, occhi arrossati, arrossamento degli occhi	Les yeux rouges
Painful eyes	Dolor en los ojos	Schmerzende augen, augenschmerzen, augenbrennen	Dolore agli occhi	Yeux douloureux
Irritated eyes	Irritación de los ojos	Gereizte augen,	Occhi irritati, irritazione degli occhi	Yeux irrités
Mouth ulcers	Úlceras en la boca	Geschwüre im mund	Ulcere della bocca, ulcerazioni della bocca, ulcere del cavo orale, ulcerazioni del cavo orale	Ulcères de la bouche
Mouth sores	Úlceras de boca, llagas en la boca, ampollas en la boca	Wunde stellen im mund	Lesioni della bocca, infiammazione della bocca, ferita alla bocca	Plaies dans la bouche
Canker sores	Aftas, llagas	Aphten	Afte, afta	Aphtes
Headache	Dolor de cabeza, dolor en los sesos	Kopfschmerzen	Mal di testa, dolore alla testa, cefalea	Mal de crâne

Migraine	Migraña, cefalea	Migräne	Emicrania	Migraine
Rash, rashes	Erupción, erupciones, exantema	Hautausschlag, ausschlag	Eruzioni cutanee, puntini rossi sulla pelle, sfogo cutaneao	Éruption cutanée, des éruptions cutanées
Pyoderma gangrenosum	Pioderma gangrenoso	Pyoderma gangraenosum	Pioderma gangrenoso	Pyoderma gangrenosum
Skin erythema	Eritema cutáneo	Hautrötung	Eritema cutaneo, irritazione cutanea, arrossamento della pelle	Érythème cutané
Fever	Fiebre, quebranto, febrícula, calentura	Fieber	Febbre, iperpiressia	Fièvre
High temperatures	Altas temperaturas	Hohe temperaturen	Rialzo della temperatura	Températures élevées, avoir de la température
Sweat, sweating	Sudor, sudoroso, sudar	Schweiß, schwitzen	Sudore, sudorazione	La sueur, la transpiration
Vomiting, vomit, vomited	Vómito, vómito, potar, devolver	Erbrechen, erbrechen, erbrach	Vomito, vomitato, vomitare	Vomissements, vomissements, vomit
Throwing up, throw up, threw up	Vomitar, vomitar, vomitó	Werfen, werfen, warf	Vomitare, rimettere	Vomir, vomir, dégueuler
Nausea, nauseous, nauseated	Náuseas, ganas de vomitar o de potar	Übelkeit, übelkeit, ekelte	Nausea	Des nausées, des nausées, des nausées, nauséeux, nauséeuses
Feel sick	Sentirse enfermo, sentirse mal	Krank fühlen	Sentirsi male, sentirsi malato,	Se sentir malade, se sentir mal

No appetite, loss of appetite	Falta de apetito, pérdida de apetito, inapetente, sin hambre	Kein appetit, appetitlosigkeit	Senza appetito, mancanza di appetito, inappetenza, anoressia, perdita di appetito, inappetente, anoressico	Pas d'appétit, perte d'appétit, plus d'appétit
Fatigue, fatigued	Cansancio, fatiga, fatigado, agotado, astenia	Müdigkeit, ermüdete, schlappheit	Astenia, astenico, affaticamento, affaticato, stanchezza, spossatezza, stanco, spossato	Fatigue, fatigué
Tired	Cansado	Müde	Stanco	Fatigué, épuisé, vidé
Palpitate, palpitation	Palpitar, palpitaciones	Palpitate, palpitationen, herzklopfen, stolpern, herzrhythmusstörungen	Palpitare, palpitazioni	Palpiter, palpitation
Racing heart	Aceleración del ritmo cardíaco	Rasendes herz	Battito accellerato	Coeur qui palpite
Beating heart	Palpitaciones en el corazón, corazón acelerado	Schlagendes herz, herzschlag	Batticuore, battiti ectopici	Cœur battant, cœur qui s'emballe, cœur qui bat trop fort
Fast heart rate	Frecuencia cardíaca rápida	Herzrasen, herzklopfen	Frequenza cardiaca accelerata, incremento frequenza cardiaca	Rythme cardiaque rapide
Tachycardia	Taquicardia	Tachykardie	Tachicardia	Tachycardie
Heart flutter	Aleteo del corazón	Herzflimmern, vorhofflimmern	Fibrillazione	Flutter cardiaque
Hard to breathe	Dificultad para respirar, falta de aire	Schwer zu atmen, luftnot, atembeschwerden, dyspnoe, lufthunger, atemlosigkei	Difficoltà nella respirazione, difficoltà a respirare, respiro difficoltoso	Difficile de respirer, difficultés à respirer

Shortness of breath	Dificultad para respirar, falta de aire, falta de aliento	Kurzatmigkeit	Fiato corto, mancanza di fiato	Essoufflement
Dyspnoea	Disnea	Dyspnoe	Dispnea	Dyspnée
Arrythmia	Arritmia	Arrythmia	Aritmia	Arythmie
Dehydration, dehydrated	Deshidratación,-deshidratado	Dehydration, dehydrated	Disidratazione, disidratato	Déshydratation, deshydraté
Thirst, thirsty	Sed, sediento	Durst, durst	Sete, assetato	Soif, avoir soif
Anxious, anxiety	Ansiedad, ansioso	Ängstlich, angst	Ansioso, ansia	Anxieux, l'anxiété
Nervous	Nervioso	Nervös	Nervoso, agitato	Nerveux
Insomnia	Insomnio	Schlaflosigkeit, schlafstörungen	Insonnia	Insomnie
Can't sleep	No puede dormir	Kann nicht schlafen, schlaflosigkeit	Non riesce a dormire	Ne peut pas dormir
Can't fall asleep	No puede conciliar el sueño	Kann nicht einschlafen	Non riesce ad addormentarsi	Ne peut pas s'endormir, du mal à s'endormir
		Geschwürbildende darmentzündung		
Blood mucilaginous consistency		Blut-schleimiger konsistenz		
Chest pain		Brustschmerzen, herzschmerzen		
**Ontology Building Words[b]**				

English	Spanish	German	Italian	French
Stomach	Estómago	Magen	Stomaco	Estomac
Belly	Vientre, abdomen, barriga	Bauch, abdomen	Pancia	Ventre
Tummy	Barriguita, tripa	Bäuchlein	Pancia	Bide
Gut		Darm		
Body	Cuerpo	Körper	Corpo	Corps
Eye	Ojo	Auge	Occhio	Œil
Mouth	Boca	Mund	Bocca	Bouche
Back	Espalda	Zurück	Schiena	Dos
Head	Cabeza	Kopf	Capo, testa	Tête, crane
Skin	Piel	Haut	Pelle	Peau
Joint	Articulación	Joint	Articolazione	Articulations
Toilet		Toilette, WC		
Stool	Heces, deposición	Schemel	Feci	Selles
Bowel movement	Defecación, evacuación, deposición, movimientos intestinales	Stuhlgang, stuhldrang	Evacuazione, defecazione	Défécation
BM	BM	BM	BM	BM

Poop	Caca, pupú, popó, excremento	Poop	Cacca	Caca
Poo		Poo	Cacca	Caca
Shit	Mierda	Scheisse, hinterlassenschaft, kot, stuhl, notdurft, exkrement, haufen, kacke, scheiße, aa, kacka, dejekt, fäzes, fäkalien	Merda	Merde
Blood, bloody, bleeding, bled	Sangre, con sangre, sangrado	Blut, blutig, bluten	Sangue, sanguinoso, sanguinolento, sanguinante, sanguinamento, sanguinava, ha perso sangue	Sang, saignant, saignement, saigné
Mucus	Moco	Schleim	Muco	Mucus
Pus	Pus	Stellen	Pus, materiale purulento	Pus
Pain, painful	Dolor, doloroso	Schmerz, schmerzhafte	Dolore, doloroso	La douleur, douloureux
Ache	Dolor	Schmerzen	Dolore	Mal, maux
Joint	Articulación	Joint	Articolazione	Articulations, articulation
Sore	Doloroso	Wunde, geschwür, weh	Doloroso, infiammato	Douloureux, endolori
Ulcer	Úlcera	Ulcera, ulcus, ulzeration, geschwür	Ulcera	Ulcère
Appetite	Apetito	Appetit	Appetito	Appétit
Heart	Corazón	Herz	Cuore	Cœur

Heartbeat	Latido del corazón, palpitación	Herzschlag	Battito cardiaco	Battement de coeur
Breath	Aliento	Atem	Respiro	Souffle
Breathe, breathing	Respirar, respiración	Atmen, atmen	Respirare, respirazione	Respirer, respiration
Sleep, sleeping, slept	Sueño, dormir, dormido	Schlaf, schlafen, schlief, schlappheit	Sonno, dormire, dormiente, dormiva, stava dormendo	Dormir, dormir, dormi, endormi
Pain management		Schmerztherapie		
Weight loss		Gewichtsverlust		

[a] Included combinations of terms (eg, bloody poop, blood in stool).

[b] The following terms were not searched for individually but paired (eg, bloody poop, blood in stool) to explore all potential variations for translation purposes.

Supplementary Figure 1. The top 10 ranked flare-related topics discussed by patients with UC in (A) the United States and (B) European countries.

A

	Discussion Topic (in decreasing order of rank)	Total Volume	Unique Authors	Negative Sentiment	Betweenness Centrality	Recency
1	Dietary recommendations	High	High	Moderate	Low	High
2	Experiences with mesalamine and other oral/rectal formulations	High	High	Low	Low	High
3	Medical test results and experiences	Moderate	High	Very low	Moderate	Low
4	Immune system and gut bacteria association	Moderate	Low	High	Moderate	Moderate
5	Mental health management	Moderate	Low	Very high	Very low	Very low
6	Bloody stool	Low	Moderate	Moderate	Moderate	Low
7	Tapering off drugs	Low	Moderate	Moderate	High	Very low
8	Comparing severity of flares	Low	Moderate	Moderate	Moderate	Low
9	Urgency and frequency	Very low	Moderate	Moderate	High	Very low
10	Experiences with budesonide	Very low	Very low	Moderate	High	Moderate

Heatmap Legend

Low volume – High volume

Low number of unique authors – High number of unique authors

Low negative sentiment – High negative sentiment

Low centrality – High centrality

Low recency – High recency

B

	Discussion Topic (in decreasing order of rank)	Total Volume	Unique Authors	Negative Sentiment	Betweenness Centrality	Recency
1	Dietary recommendations	High	High	Very low	Low	Moderate
2	Peer support and mental health	High	High	Moderate	Moderate	Moderate
3	Impact of UC on quality of life	High	High	Moderate	Moderate	Moderate
4	Medical test results and experiences	High	Moderate	Very low	Moderate	Very low
5	Abdominal pain	Moderate	Moderate	Very high	High	Very low
6	Blood and mucus in stool	Moderate	Moderate	Low	Moderate	Very low
7	Experiences with corticosteroid treatment	Moderate	Low	High	High	Very low
8	Urgency and frequency	Moderate	Low	Low	Very low	High
9	Treatment side effects	Low	Low	Moderate	Low	Moderate
10	Impact of antibiotics on UC flares	Very low	Very low	Moderate	High	High

Heatmap Legend

Low volume — High volume

Low number of unique authors — High number of unique authors

Low negative sentiment — High negative sentiment

Low centrality — High centrality

Low recency — High recency

Flare-related posts, including those from the same author pre- and postflare, were identified from posts written by patients with UC on 8 online public forums in 6 different countries due to the inclusion of keywords. Posts were stratified by region (United States and European countries

[France, Germany, Italy, Spain, and the United Kingdom]) and topics were identified and ranked by a combined weighted score of volume (40%), unique authors (20%), negative sentiment (15%), betweenness centrality (15%), and recency (10%). UC, ulcerative colitis.

References

1. Law EH, Auil MJ, Spears PA, Berg K, Winnette R. Voice analysis of cancer experiences among patients with breast cancer: VOICE-BC. *J Patient Exp.* 2021;8:23743735211048058.
2. Farrar M, Lundt L, Franey E, Yonan C. Patient perspective of tardive dyskinesia: results from a social media listening study. *BMC Psychiatry.* 2021;21(1):94.
3. Silverberg JI, Feldman SR, Smith Begolka W, et al. Patient perspectives of atopic dermatitis: comparative analysis of terminology in social media and scientific literature, identified by a systematic literature review. *J Eur Acad Dermatol Venereol.* 2022;36(11):1980-1990.
4. Blondel VD, Guillaume J-L, Lambiotte R, et al. Fast unfolding of communities in large networks. *J Stat Mech.* 2008;2008:P10008.
